# Annotations

**Published:** 1906-12-01

**Authors:** 


### ANNOTATIONS.

### Influenza.

We understand that a not unusual, though unwelcome visitor of this time of year, in the form of influenza, has made its appearance in our midst in a rather pronounced manner. The moist warm weather we have so far experienced may not improbably be largely responsible for this. Such meteorological conditions are undoubtedly, according to previous observations, favourable to the development and spread of the influenza bacillus. We do not know whether the bacteriologists have yet busied themselves with investigations as to the nature of the microbe, which is now sporting itself amidst the community, whether it is the true influenza bacillus of Pfeiffer or some modification of that species. Clinically the disease now prevalent lacks the severity and the variety of form so characteristic of the epidemic type. The cases so far have followed a fairly stereotyped course. The onset is much more sudden than usually occurs in sporadic cases, and is characterised by the usual fever (temperature generally 102° to 104°) accompanied by headache and pains in the back and limbs, and sometimes by vomiting. These symptoms yield to treatment within the first two or three days, but the temperature remains at about 100° for some time longer, and is accompanied by a profuse nasal catarrh, followed by a more or less troublesome catarrhal affection of the bronchial tubes. Gastro-intestinal symptoms are fairly common, notably the vomiting of onset already referred to, and we understand that these symptoms form a specially troublesome feature of the disease in certain parts of the country. It is extremely infectious, and when once it has gained access to a household it rapidly makes victims of many of the members in succession.

### School Lessons in Hygiene and Temperance.

Very general sympathy will be felt with the motives of the distinguished medical deputation which recently waited on Mr. Birrell at the Board of Education to urge the compulsory teaching of hygiene and temperance in the elementary schools of the country. The weight of authority presented to the Minister for Education cannot be disputed, and the objects aimed at are without question. But to have value the question must be presented in a concrete form, and it is by no means certain that distinguished physicians and surgeons are the best authorities either on what it is possible to teach young children on these subjects or on the most appropriate methods of instruction. Even on some elementary problems in hygiene and temperance there are differences of opinion among experts, and much discretion will be necessary in introducing such matters into the school-room. Some of the most ardent supporters of the objects discussed by the deputation are not free from the suspicion of "faddism," and they themselves must at least allow that their special views are in advance of public opinion. Alcohol, in such relationship, is a particularly thorny subject, and the pert youngster abounding in the decisive dogmas of a recent "temperance" lesson might well prove a formidable critic to the habits of many households. Mr. Birrell, in replying to the deputation, discussed the whole position in an eminently sane and reasonable speech, and whatever differences of opinion may exist on other points, most medical men will agree with the substance of his remarks. It is morally certain that no sensible effect will be produced on the minds of the children except through the medium of their teachers. Here is the spring through which the whole course and character of the stream may be influenced. Therefore the vital point is to get efficient teaching of the principles of health preservation introduced into the curriculum of the training colleges. If this is effected and is framed on sound lines the precepts, and we hope the example, of the elementary school teachers will gradually become operative in the lives of their scholars.

### Lunacy and Marriage.

The increase in the proportion of lunatics to population is a favourite subject of debate wherever sociologists are gathered together. Perhaps these gentlemen, especially when they happen to be professional alienists, whose lives are largely spent among the insane, incline to take too gloomy a view of the subject. The word "insanity" has had its scope enlarged of late years. Formerly senile decay was not so described, and the idiot or "natural" which every village boasted was not counted among the nation's lunatics. In those days the treatment of the insane in asylums was so harsh that those who loved their kin of weak mind bore all things, risked all things, from them rather than send them to such a place. And those who were sent never came out. The asylum, where they experienced harsh treatment from attendants who had neither knowledge nor kindness of heart, where the food was coarse, even if not actually bad; where the common use of physical restraint acted injuriously on the over-strained nerves and deranged mind, was indeed a living grave. Now all these things are changed, and everything that care and comfort can provide is lavished on the lunatic. The result is that many cases are, or seem to be, cured. The patients go out, free to take any part they like in the business of the world, which world, not showing them the same consideration that they had found in their retirement, again in many cases drives them off their balance. Some cases return to the asylum again and again, are "cured" and relapse once more. But in the meantime they are absolutely free, so far as the law goes, to marry and become parents. And that is why, in the statistics of lunacy, we so often find the cause of the mental failure set down as heredity. We should be loth to say that everyone whose mind has once been temporarily unhinged by grief, anxiety, or physical pain, is, therefore, doomed to celibacy, that man or maid whose father or mother's mental health once broke down should never marry. But at least the risks should be better known than they are at present, and some restriction might be put on the marriage of those whose record of mental health is so bad as to promise a heritage of insanity to their children.

